# Spatial and Temporal Distribution of Tuberculosis in the State of Mexico, Mexico

**DOI:** 10.1100/2012/570278

**Published:** 2012-07-31

**Authors:** Adrian Zaragoza Bastida, Marivel Hernández Tellez, Lilia P. Bustamante Montes, Imelda Medina Torres, Jaime Nicolás Jaramillo Paniagua, Germán David Mendoza Martínez, Ninfa Ramírez Durán

**Affiliations:** ^1^Facultad de Medicina, Universidad Autónoma del Estado de México, 50180, Toluca, MEX, Mexico; ^2^Centro Interamericano de Recursos del Agua, Universidad Autónoma del Estado de México, 50200, Toluca, MEX, Mexico; ^3^Facultad de Medicina Veterinaria Zootécnia, Universidad Autónoma del Estado de México, 50200, Toluca, MEX, Mexico; ^4^Departamento de Producción Agrícola y Animal, Universidad Autónoma Metropolitana, 04960, México, DF, Mexico

## Abstract

Tuberculosis (TB) is one of the oldest human diseases that still affects large population groups. According to the World Health Organization (WHO), there were approximately 9.4 million new cases worldwide in the year 2010. In Mexico, there were 18,848 new cases of TB of all clinical variants in 2010. The identification of clusters in space-time is of great interest in epidemiological studies. The objective of this research was to identify the spatial and temporal distribution of TB during the period 2006–2010 in the State of Mexico, using geographic information system (GIS) and SCAN statistics program. Nine significant clusters (*P* < 0.05) were identified using spatial and space-time analysis. The conclusion is that TB in the State of Mexico is not randomly distributed but is concentrated in areas close to Mexico City.

## 1. Introduction

Tuberculosis (TB) is an infectious disease, generally chronic, and caused by a group of bacteria: *Mycobacterium tuberculosis, M. africanum, M. bovis, M. microti* and* M. canettii* [1]. The main transmission is from person to person by microdrops generated by coughing or sneezing of a person with active TB [1, 2].

TB is one of the oldest human diseases that still affects large population groups, mainly in marginal areas and comprising vulnerable groups impacted by extreme poverty, malnutrition, and crowded housing. These groups are prone to infection by the tuberculosis bacilli and to acquiring active TB [2, 3].

In Mexico, the National Population Commission (CONAPO) considers a marginal index based on a census of localities according to the global impact of several total measurements or items, such as lack of education (illiteracy, incomplete grade school) and inadequate housing conditions (without water, drainage, bathrooms, electricity, refrigerator [4], and other basic household equipment, houses with earth floors and crowded living conditions). These items can be used to identify geographic areas with greater marginalization (socioeconomic development) and a greater risk from TB infection.

The World Health Organization (WHO) reported in 2010 that there were an estimated 9.4 million incident cases (range 8.9 million–9.9 million) of TB globally, equivalent to 137 cases per 100,000 population, and that 1.1 million of those cases also tested positive for human immunodeficiency virus (HIV). The mortality of HIV-negative patients with TB was estimated at 1.3 million, this being equivalent to 20 deaths per 100,000 people. The incidence of TB patients in Asia was 55% and 30% in Africa; smaller proportions of cases occurred in the Eastern Mediterranean Region (7%), the European Region (4%), and the Americas Regions (3%) [5].

In Mexico, 18,848 TB cases (in all clinical forms) were reported in 2010, which is a rate of 16.77 per 100,000 habitants. Of all the cases reported, 15,384 or 13.7 per 100,000 corresponded to lung TB, the mortality rate was 2.14 per 100,000 (in all clinical forms) [6], and the incidence rate was higher in the 60-year-old or 60+-year-old groups of patients, and males had a 1.5 times higher rate than females [3].

In the federal entities, TB presents a well-defined concentration pattern. High incidence rates are present in the western states and the Gulf of Mexico, and lower rates are found in the central states. Tamaulipas and Baja California have double the national rate (13.5 per 100,000 inhabitants). In these states, together with the Veracruz, Chiapas, Nuevo León, Jalisco, Sinaloa, Nayarit, Guerrero, Sonora, Oaxaca, and Chihuahua states, are concentrated 70% of all the identified cases of TB in the country [3].

At present, geographic information systems (GISs) are among the most useful tools in epidemiology, as they can be used to identify geographical areas and population groups with a higher risk of sickness or premature mortality and which therefore require higher preventive care or health information and monitoring of diseases in time and space [7, 8].

Spatial cluster of a disease grouping is one of the most important techniques in epidemiology. In 1995, Kuldorff and Negarwalla developed a new space statistics method called SCAN for the detection and cluster inference in time-space under the likelihood ratio (LLR) hypothesis [8].

SCAN statistics is a spatial-temporal analysis defined by a large number of transposed cylinders; the circular base defines the geographic area with a radius that can vary from zero to a larger distance influenced by the percentage of the population at risk, while the height of the cylinder represents the temporal parameter. The LLR was calculated for each cylinder. The cylinder with the highest LLR was considered to constitute the most probable cluster. A Monte Carlo simulation was used to calculate the *P* value [9].

In the case of TB, various researchers have used GIS to study this infectious disease. Moonan et al. [10] used GIS to identify the geographic locations of TB transmission and incidence in the United States of America during 1993 to 2000. In India, Tiwarin et al. [11] carried out a geospatial investigation of TB occurrence in the Almora district using GIS and the SCAN statistics program. Nunes [12] in Portugal detected spatial and temporal clusters during 2000–2004 by using SCAN. The above-mentioned authors agree that GIS and SCAN are useful tools for vigilance against TB.

The SCAN statistics program has been used worldwide by various researchers with different lengths of search windows: the most frequently used are 25% and 50% of the population [11]. Sabel et al. [13] added some health determinants in their model, such as age, gender, and margination, as covariants as a form of correction or data adjustment. 

The objective of this research was to use the spatial statistics program SCAN and GIS to identify the spatial and temporal distribution of TB during 2006 to 2010 and to determine the geographic locations of higher transmission and incidence of TB in the State of Mexico, Mexico.

## 2. Materials and Methods

### 2.1. Study Location

**Figure 1:**
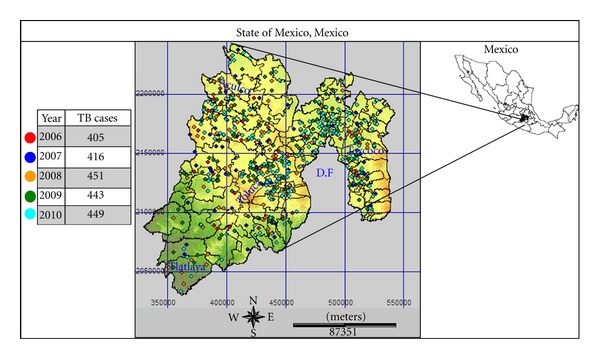
Location and geographic distribution of tuberculosis cases during 2006–2010 in the State of Mexico.

This study used as its research area the State of Mexico, located in the centre of Mexico at 98°35′30′′–100°37′00′′ west longitude and 18°21′15′′–20°17′00′′ north latitude, and occupying 22,499.95 km^2^, which represents 1.1% of the national territory. It is divided into 125 municipalities that include 4, 341 localities (Figure 1). 

According to the II Conteo de Población and Vivienda 2005 (II Population and Housing Census 2005), the State of Mexico had 14,007,495 inhabitants, representing 13.6% of the entire Mexican population and making it the most populated state.

### 2.2. Data Collection 

#### 2.2.1. Population Data

In accordance with the Sistema Nacional de Vigilancia Epidemiológica (Epidemiology Vigilance National Surveillance), tuberculosis cases were reported from 499 localities during the period 2006–2010. Cases in each locality were categorized using a marginalization index [14]: Category 1: very low; Category 2: low; Category 3: medium; Category 4: high; Category 5: very high. The number, age and gender of the inhabitants in each locality were obtained from the II Population and Housing Census 2005 by the “Instituto Nacional de Estadística y Geografía” (INEGI) (National Institute of Statistics and Geography) [15]. Age was classified into five categories: Category 1: 0–4 years old; Category 2: 5–14 years old; Category 3: 15–17 years old; Category 4: 18–59 years old; Category 5: 60 and 60+ years of age. The data for gender was combined with the age categories, and therefore 10 groups of age-gender were created to describe the population of each locality.

#### 2.2.2. Tuberculosis Case Data

The collected cases were diagnosed at several hospitals and healthcare centres and their demographic characteristics were reported to the TB National Registry during 2006–2010. Marginality was obtained from the 2005 marginality index [14] for the locality where each TB case was found. For the statistical strategy, the covariables were age-gender and margination. 

#### 2.2.3. Geographic Localization Data

For data analysis, locality with the TB case was used as a geographic unit. The georeference came from the latitude-longitude projection system obtained from the Instituto de Geografía Estadística y Catastral del Estado de México (IGECEM) (Institute of Statistical Geography and Tax Revenue of the State of Mexico) [16].

### 2.3. Statistical and Geographic Analysis

#### 2.3.1. TB Cluster Detection and Identification

Determination and identification of TB clusters were carried out with the statistics spatial SCAN program and calculated with the SaTScan (V8.0) computer package that obtains statistical significance and the approximate cluster localization. The SaTScan program requires three files for initiation: cases, population, and geography localization files. A Poisson probability model was used with a 25% and 50% search window for high rates: age, gender and, margination index were the covariables.

#### 2.3.2. Geographic Analysis

For the graphic cluster representation, a map was drawn based on the information obtained with the SCAN and the IDRISI Taiga software. A factorial format map of the State of Mexico obtained from the IGECEM and a Lansat 7 satellite medium infrared colour image at bands 2, 4, and 7 were used [17].

## 3. Results

**Figure 2:**
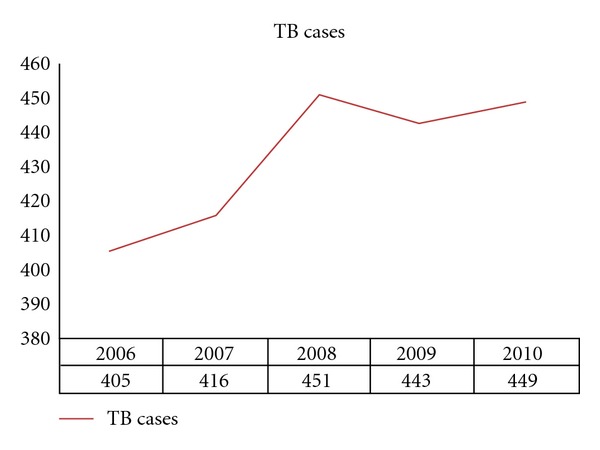
Frequency of TB cases in the State of Mexico during the years 2006 to 2010.

There were 2,164 human TB cases detected from 2006 to 2010, distributed in 499 localities and in 125 municipalities. The municipalities with more than 100 TB cases are Ecatepec de Morelos with 365 (16.9%) cases; Netzahualcóyotl with 172 (7.9%); Naucalpan de Juárez with 153 (7%); Tlalnepantla de Baz with 123 (5.7%); Toluca county with 109 cases (5%). The distribution of the 2,164 cases is shown in Figure 2 with an increment from 405 cases in 2006 up to 449 in 2010.

As mentioned in the methodology section, the 10 “age-gender-margination” groups or categories were found in 499 locations with TB cases. A database with 4990 groups was created. Two analyses were performed using the collected information: the first analysis was spatial and the second was a space-time analysis.

### 3.1. Spatial Analysis

A search window with a maximum length of ≤50% population with high TB rates was used. A primary cluster and five secondary clusters were identified. The centre of the primary cluster was located in the municipality of Valle de Chalco Solidaridad in the locality of Santa Cruz. The relative risk (RR) was 337.68 with 14 TB cases *versus* the 0.04 expected cases. The second cluster was formed of the municipalities of Acolman, Atenco, Chiautla, Chiconcuac, Ecatepec de Morelos, Papalotla, Tecámac, Teotihuacán, Tepetlaoxtoc, Texcoco, and Tezoyuca. The RR was 1.68 with 423 TB cases and the expected RR was 282.2 cases. The third cluster, in the Atizapán de Zaragoza, Villa Nicolás Romero, and Cuautitlán Izcalli municipalities, had a 215.58 RR with seven TB cases compared to 0.03 expected cases. The 10.75 RR for the fourth cluster with 20 TB cases, located in Huixquilucan and Lerma municipalities, had an expected number of cases of 1.88.

Cluster 5, located in the municipalities of Tlalnepantla de Baz, Tultitlán, Apizapán de Zaragoza, and Cuahutitlán Izcalli, was found to have a 4.39 RR with 38 TB cases compared with the 8.77 expected cases.

The last identified secondary cluster, located in the Tlalnepantla de Baz, Jilotzingo, Atizapán de Zaragoza, and Naucalpan de Juaréz, showed a 10.24 RR with eight TB cases against the 0.78 expected cases. 

**Figure 3:**
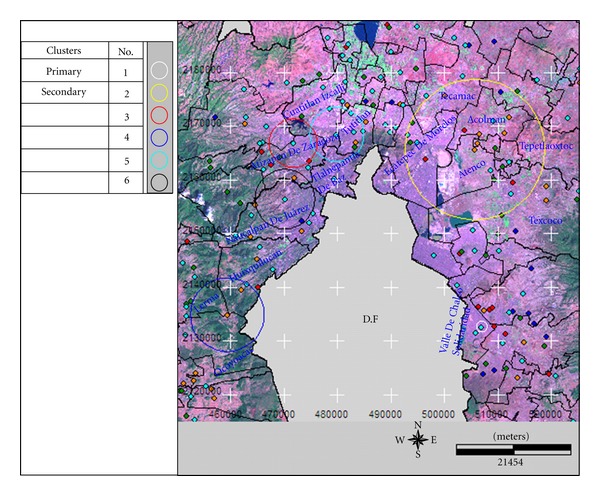
Geography of the identified high TB rate clusters in the State of Mexico detected with a spatial analysis and a search window maximum length of ≤50% and ≤25% population, adjusted for age-gender-margination.

A maximum length of ≤25% search window was used for a population with high TB rates and the results of the analysis were similar to those obtained with a ≤50% search window. General information about the identified clusters is shown in Table 1, and their geography is shown in Figure 3.

### 3.2. Space-Time Analysis

A primary cluster was identified using a search window maximum of ≤50% population with high TB rates. The cluster centre was located in the Tultitlan, a municipality formed of the Tlalnepantla de Baz, Cuautitlán, Cuautitlán Izcalli, and Atizapán de Zaragoza municipalities. The relative risk (RR) during the 2007–2009 period was 6.22 with 27 TB cases and 4.39 expected cases. Secondary clusters were not identified.

A search window maximum length of ≤25% population was used to identify three significant clusters. The primary cluster was located in the Valle de Chalco Solidaridad, Ixtapaluca, and a small part of Chalco. The RR was 81.08, with 16 TB cases during the period 2008–2006, compared with 0.2 expected cases. 

**Figure 4:**
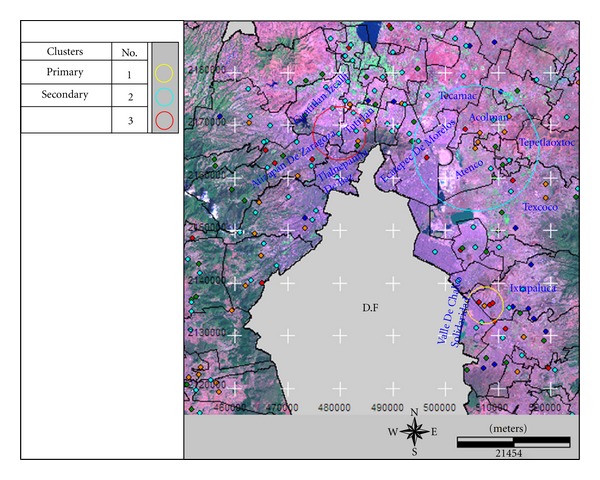
Geographic locations of the identified clusters with high rates using a space-time analysis and a search window maximum length of ≤50% and ≤25% population, adjusted for age-gender-margination.

Two secondary clusters were identified. One was similar to that described above using the ≤50% population search window. The other was formed by 11 municipalities: Acolman, Atenco, Chicuautla, Chiconcuac, Ecatepec de Morelos, Papalotla, Tecamac, Teotihuacan, Tepetlaoxtoc, Texcoco, and Tezoyuca. The RR was 2.03 with 267 cases of TB during the years 2006–2008 against expected cases of 140.50. General information about the identified clusters is shown in Table 2 and their geography in Figure 4.

## 4. Discussion

In the field of epidemiology one of the most important analyses is the detection in space-time of a disease cluster, since a non-control-spreading of the disease takes place and a social or economic factor favours cluster formation. The use of GIS plus spatial statistics has been used to determine spatial distribution patterns of various infectious and noninfectious diseases [18–22]. SCAN, available in the SaTScan V8 software, has been used worldwide to detect various disease clusters, including TB.

GISs plus molecular diagnosis techniques have been used to locate TB clusters and to identify the *Mycobacterium* responsible for the outbreak [10]. Frequently, however, it is not possible to obtain a detailed and complete analysis since the information is mainly based on a positive bacilloscopy.

However, this study obtained data on TB according to age, gender, and marginality index, representing valuable information since the highest rate of this disease occurs in adults of 60 and 60+ years of age [3], in males 1.5 to 1 rate and in marginal groups [2].

With the SCAN statistical results and a 25% and 50% search window population and a spatial analysis (*P* ≤ 0.05), six clusters were identified, showing that TB is not randomly distributed in the State of Mexico but in clusters in a spatial pattern. In similar studies by Navaet al. [23] in Acapulco, Mexico, using a different spatial method, clusters were not found. We used two search windows (50% and 25%) in order to find small clusters but identification differences were not seen in cluster identification. These results were in agreement with those reported by Tiwarin et al. [11]. 

The six identified clusters are located close to and around Mexico City (Federal District), the largest city in the Mexico. The highest incidence rates of TB are found in urban zones [24] in comparison with rural areas. Our results agreed with those reported by Moonan et al. [10]. They found a strong association between the strains of TB clusters and the distance to the centre of urban zones. 

Three TB clusters were identified with spatial and temporal analysis using the same search windows; two of these clusters grouped the TB cases from the 2006 to 2008 period, and the cases from 2007 to 2009 were in the third cluster. The three clusters all contained the 2008 TB cases, making it the year with the highest incidence.

Of the three identified clusters, only one is in agreement with those identified with the spatial-only analysis, but the others are located in areas close to the spatial clusters.

The results of the present study are of great importance in TB epidemiological surveys in the State of Mexico. The presence of clusters is valuable to the healthcare system since strategy can be revised accordingly in those areas containing TB clusters.

## 5. Conclusions

TB is spatially clustered in the State of Mexico, Mexico. Nine clusters were identified using two types of analysis: spatial and spatial-temporal. Such clusters were mainly found in Valle de Chalco Solidaridad, Atenco, Ecatepec de Morelos, Tlalnepantla de Baz, Acolma, Tultitlan, Cuautitlán Izcalli, Atizapán de Zaragoza, Huixquilucan, and Naucalpan de Juárez, all municipalities that are very close to Mexico City (Federal District).

## Figures and Tables

**Table 1:** TB clusters with a high RR identified in the State of Mexico using a space analysis and a search window maximum length of ≤50% and ≤25% population, adjusted for age-gender-margination.

Cluster	Loc.^a^	Centre coordinates	Radius (km)	Obs^b^	Exp^c^	RR^d^	LLR^e^	*P*
Primary cluster								
1	1	19.287 N, 98.933 W	**0**	**14**	0.04	337.68	67.5	0.001
Secondary clusters								
2	23	19.585 N, −98.945 W	13.11	423	282.20	1.62	35.8	0.001
3	3	19.596 N, −99.269 W	4.48	7	0.03	215.58	30.6	0.001
4	3	19.306 N, −99.386 W	6.88	20	1.88	10.75	29.3	0.001
5	5	19.610 N, −99.191 W	5.08	38	8.77	4.39	26.7	0.001
6	4	19.501 N, −99.284 W	5.57	8	0.78	10.24	11.4	0.001

^a^Locations with TB cases, ^b^Observed number of cases in cluster, ^c^Expected number of cases in cluster, ^d^Relative risk of the cluster, ^e^Log likelihood ratio.

**Table 2:** A cluster with high rates of tuberculosis in the State of Mexico was detected with a spatial-temporal analysis and a search window maximum length of ≤50% and ≤25% risk population, adjusted for age-gender-margination.

Cluster	Loc.^a^	Centre coordinates	Radius (km)	Year	Obs^b^	Exp^c^	RR^d^	LLR^e^	*P*
Primary cluster									
1	6	19.315 N, −98.928 W	3.49	2006–2008	16	0.2	81.08	54.467248	0.001
Secondary clusters									
2	19	19.585 N, −98.945 W	11.93	2006–2008	267	140.59	2.03	48.880307	0.001
*3	5	19.610 N, −99.191 W	5.08	2007–2009	27	4.39	6.22	26.578700	0.001

^a^Locations with TB cases, ^b^Observed number of cases in cluster, ^c^Expected number of cases in cluster, ^d^Relative risk of the cluster, ^e^Log likelihood ratio.

^∗^Cluster detected with a search window maximum length of ≤50% risk population.

## References

[1] Lawn SD, Zumla AI (2011). Tuberculosis. *The Lancet*.

[2] Baker MG, Venugopal K, Howden CP (2011). *Household Crowding and Tuberculosis*.

[3] Secretaría de Salud *Programa de Acción Específico 2007–2012 Tuberculosis*.

[4] http://www.conapo.gob.mx/index.php?option=com_content&view=article&id=46&Itemid=194.

[5] World Health Organization (WHO) (2010). *Global Tuberculosis Control*.

[6] Secretaría de Salud (2010). *Dirección General Adjunta del Programas Preventivos del Centro Nacional de Vigilancia Epidemiológica y Control de Enfermedades (CENAVECE)*.

[7] Martinez R, Vidaurre M, Najera P, Loyola E, Castillo C (2001). SIG-Epi uso de sistemas de información geográfica en epidemiología y Salud Pública. *Boletín Epidemiológico, Organización Panamericana de la Salud*.

[8] Kulldorff M, Nagarwalla N (1995). Spatial disease clusters: detection and inference. *Statistics in Medicine*.

[9] Kulldorff M, Mostashari F, Duczmal L, Yih WK, Kleinman K, Platt R (2007). Multivariate scan statistics for disease surveillance. *Statistics in Medicine*.

[10] Moonan PK, Bayona M, Quitugua TN (2004). Using GIS technology to identify areas of tuberculosis transmission and incidence. *International Journal of Health Geographics*.

[11] Tiwari N, Adhikari CMS, Tewari A, Kandpal V (2006). Investigation of geo-spatial hotspots for the occurrence of tuberculosis in Almora district, India, using GIS and spatial scan statistic. *International Journal of Health Geographics*.

[12] Nunes C (2007). Tuberculosis incidence in Portugal: spatiotemporal clustering. *International Journal of Health Geographics*.

[13] Sabel CE, Wilson JG, Kingham S, Tisch C, Epton M (2007). Spatial implications of covariate adjustment on patterns of risk: respiratory hospital admissions in Christchurch, New Zealand. *Social Science and Medicine*.

[14] http://www.conapo.gob.mx/index.php?option=com_content&view=article&id=46&Itemid=194.

[15] http://www.inegi.org.mx/sistemas/consulta_resultados/iter2005.aspx?c=27436&s=est.

[16] http://igecem.edomex.gob.mx/descargasgeograficas.htm.

[17] http://igecem.edomex.gob.mx/descargasgeograficas.htm.

[18] Elias J, Harmsen D, Claus H, Hellenbrand W, Frosch M, Vogel U (2006). Spatiotemporal analysis of invasive meningococcal disease, Germany. *Emerging Infectious Diseases*.

[19] Bonilla RE (2006). Distribución espacio-temporal de la fiebre dengue en Costa Rica. *Población y Salud en Mesoaméroca*.

[20] Oeltmann JE, Varma JK, Ortega L (2008). Multidrug-resistant tuberculosis outbreak among US-bound Hmong refugees, Thailand, 2005. *Emerging Infectious Diseases*.

[21] Wheeler DC (2007). A comparison of spatial clustering and cluster detection techniques for childhood leukemia incidence in Ohio, 1996–2003. *International Journal of Health Geographics*.

[22] Gregorio DI, Samociuk H, DeChello L, Swede H (2006). Effects of study area size on geographic characterizations of health events: prostate cancer incidence in Southern New England, USA, 1994–1998. *International Journal of Health Geographics*.

[23] Nava-Aguilera E, López-Vidal Y, Harris E (2011). Clustering of mycobacterium tuberculosis cases in Acapulco: spoligotyping and risk factors. *Clinical and Developmental Immunology*.

[24] Graham Barr R, Diez-Roux AV, Knirsch CA, Pablos-Méndez A (2001). Neighborhood poverty and the resurgence of tuberculosis in New York City, 1984–1992. *American Journal of Public Health*.

